# Effectiveness and Safety of the COVID-19 Vaccine in Patients with Rheumatoid Arthritis in a Real-World Setting

**DOI:** 10.3390/vaccines12060672

**Published:** 2024-06-18

**Authors:** María Torres-Rufas, Esther F. Vicente-Rabaneda, Laura Cardeñoso, Ainhoa Gutierrez, David A. Bong, Cristina Valero-Martínez, José M. Serra López-Matencio, Rosario García-Vicuña, Miguel A. González-Gay, Isidoro González-Álvaro, Santos Castañeda

**Affiliations:** 1 Hospital Universitario de La Princesa, Calle Diego de León 62, 28006 Madrid, Spain; mtrufas@salud.madrid.org; 2 Rheumatology Department, Instituto de Investigación Sanitaria de La Princesa (IIS-Princesa), Hospital Universitario de La Princesa, Calle Diego de León 62, 28006 Madrid, Spain; cristina.valmart@gmail.com (C.V.-M.); vicuna111@gmail.com (R.G.-V.); isidoro.ga@ser.es (I.G.-Á.); 3 Microbiology Department, IIS-Princesa, Hospital Universitario de La Princesa, Calle Diego de León 62, 28006 Madrid, Spain; lacado@gmail.com (L.C.); ainhoa.gutierrez@salud.madrid.org (A.G.); 4 Instituto Poal de Reumatología, Carrer de Castanyer, 15, Sarrià-Sant Gervasi, 08022 Barcelona, Spain; dabong47@gmail.com; 5 Bellvitge Campus, Universitat de Barcelona, Carrer de la Feixa Llarga, s/n, L’Hospitalet de Llobregat, 08907 Barcelona, Spain; 6 Hospital Pharmacy Department, Hospital Universitario de La Princesa, Calle Diego de León 62, 28006 Madrid, Spain; josemaria.serra@salud.madrid.org; 7 Rheumatology Department, IIS-Fundación Jiménez Díaz, Hospital Universitario Fundación Jiménez Díaz, Avenida de los Reyes Católicos, 2, Moncloa-Aravaca, 28040 Madrid, Spain; miguelaggay@hotmail.com; 8 Medicine and Psychiatry Department, University of Cantabria, 39008 Santander, Spain

**Keywords:** rheumatoid arthritis, COVID-19 vaccine, humoral response, effectiveness, safety

## Abstract

Novel mechanisms of COVID-19 vaccines raised concern about their potential immunogenicity in patients with rheumatoid arthritis (RA) undergoing immunomodulatory treatments. We designed a retrospective single-center study to investigate their effectiveness and safety in this population, analyzing data from the first vaccination program (December 2020–October 2021). Inclusion criteria were availability of post-vaccination serology and a minimum subsequent follow-up of 6 months. Binding antibody units (BAU/mL) ≥ 7.1 defined an adequate serological response. Post-vaccine COVID-19 incidence and its timing since vaccination, adverse events (AEs), and RA flares were recorded. Adjusted logistic and linear multivariate regression analyses were carried out to identify factors associated with vaccine response. We included 118 patients (87.2% women, age 65.4 ± 11.6 years, evolution 12.0 ± 9.6 years), of whom 95.8% had a complete vaccination schedule. Adequate humoral immunogenicity was achieved in 88.1% of patients and was associated with previous COVID-19 and mRNA vaccines, whereas smoking, aCCP, age, and DMARDs exerted a negative impact. Post-vaccine COVID-19 occurred in 18.6% of patients, a median of 6.5 months after vaccination. Vaccine AE (19.5%) and RA flares (1.7%) were mostly mild and inversely associated with age. Our results suggest that COVID-19 vaccines induce adequate humoral immunogenicity, with an acceptable safety profile in RA patients.

## 1. Introduction

Rheumatoid arthritis (RA) is a chronic systemic inflammatory disease that characteristically begins as a symmetrical polyarthritis of the small joints of the hands and feet, but can also affect large joints and even associate extra-articular manifestations that cause significant morbidity and mortality. RA is more common in women and represents one of the most frequent rheumatic diseases, with a prevalence of 0.9% in Spain and 0.2–1.2% worldwide [1,2].

Its etiopathogenesis is related to predisposing genetic factors (HLA-DR4 in the white race, shared epitope), which, subjected to various environmental stimuli that are not completely elucidated (continuous exposure to tobacco, certain viral infections, etc.) can trigger an immune response that initiates subclinical organic damage that, after a second impact or environmental exposure, gives rise to symptomatic joint and/or extra-articular damage. This underlying immune disorder justifies treatment with immunomodulatory drugs such as disease-modifying antirheumatic drugs (DMARDs), frequently requiring the addition of glucocorticoids (GCs) [3].

The immune system dysfunction generated by the disease itself and by its immunomodulatory treatments increases the predisposition of these patients to infections as well as their severity. For this reason, the magnitude and severity of the SARS-CoV-2 coronavirus pandemic raised concerns about its potential impact on RA patients. In the early stages of the SARS-CoV-2 pandemic, several studies suggested that the risk of the coronavirus disease 2019 (COVID-19) was 1.3–3 times higher in patients with autoimmune diseases, and that the disease itself might constitute a risk factor for infection severity related to high disease activity or to its treatment, such as rituximab (RTX) or high doses of GC [4,5,6,7]. However, later evidence in RA patients identified the same risk factors for disease severity that were commonly seen in the general population, such as advanced age and comorbidities, including cardiovascular disease (CVD), chronic obstructive pulmonary disease (COPD) or chronic kidney failure [6].

The lack of a specific treatment for COVID-19 at the beginning of the pandemic warranted the development of an effective vaccine that would prevent transmission and/or severe clinical manifestations in patients with RA. However, the clinical trials carried out for the vaccine registration did not allow conclusions to be drawn for patients with RA, as they were excluded [8,9]. Based on previous experience with influenza and pneumococcal vaccines [10,11,12,13,14], there was concern that the humoral immunogenicity of the SARS-CoV-2 vaccine in patients with RA on DMARDs therapy might be decreased. Thus, the American College of Rheumatology (ACR) published preliminary recommendations agreed on by experts, which have since been updated. These recommendations suggested temporarily stopping some DMARDs after vaccination against SARS-CoV-2 in patients with stable RA at the discretion of their attending rheumatologist [8,15]. However, the lack of scientific evidence and uniform recommendations from different societies on the guidelines to be followed at the beginning of the vaccination campaign led to the heterogeneous management of DMARDs in the peri-vaccine period.

Additionally, the quite novel mechanisms of SARS-CoV-2 vaccines, messenger RNA (mRNA) vaccines (BNT162b2 from Pfizer^®^ (Pfizer, New York, NY, USA), mRNA-1273 from Moderna^®^, (Moderna, Cambridge, MA, USA)), and adenovirus vector vaccines (AZD1222 from Astra-Zeneca^®^ (Astra-Zeneca, Cambridge, UK)), Ad.26.COV2.S from Johnson & Johnson^®^ (Johnson & Johnson, New Brunswick, NJ, USA), together with their different dosages and administration schedule, made it especially relevant to investigate the factors related to their efficacy and safety in RA [16].

In this context, we decided to investigate the impact of COVID-19 vaccines in our patients with RA. We hypothesized that COVID-19 vaccines would be safe and capable of inducing adequate humoral response in the majority of them, allowing to reduce the incidence and/or severity of SARS-CoV2 infection. Furthermore, we suspected that factors such as disease activity, age, comorbidity, treatment with DMARDs or GC, and the type of vaccine administered could have an influence on the efficacy and safety of the COVID-19 vaccine in this population [12,14,17,18].

## 2. Materials and Methods

This retrospective longitudinal study included patients with RA that had been vaccinated against the SARS-CoV-2 during the first COVID-19 vaccination campaign in 2020–2021 with subsequent follow-up under the conditions of real-world clinical practice in a tertiary hospital center for at least 6 months.

### 2.1. Objectives of the Study

#### 2.1.1. Primary Objectives

To evaluate the humoral immunogenicity induced by the SARS-CoV-2 vaccine and to investigate its safety in patients with RA.

#### 2.1.2. Secondary Objectives

To identify the factors associated with the humoral response to the vaccine and its potential adverse events and to analyze the protective effect of this vaccine against the SARS-CoV-2 infection and/or its severity.

### 2.2. Study Population

Patients with RA diagnosed according to the 1987 ACR classification criteria [19] and/or the 2010 ACR/EULAR classification criteria [20], treated in the Rheumatology Department of the Hospital Universitario de La Princesa (HUP), Madrid, Spain, who had been administered the COVID-19 vaccine between December 2020 and October 2021, had a post-vaccination control serology and a subsequent follow-up at the outpatients’ clinic for a minimum of 6 months. RA patients who did not have post-vaccination serology or whose subsequent follow-up was less than 6 months at the time of carrying out this investigation were excluded from the study.

The study protocol received approval from the Ethics Committee for Research with Medicines of the HUP (number ecl.4790). Given that this is a retrospective “non-intervention” study in the context of a research study in the academic field (final degree project), the Ethics Committee of our center considered that informed consent (IC) was not required with the commitment to the responsible use of these data in accordance with our legislation on medical research studies and data protection.

### 2.3. Analyzed Variables

Reviewing the data available in each patient’s electronic medical record, the variables listed in Table S1 (Supplementary Materials), including socio-demographic characteristics, RA and its treatment, vaccination against SARS-CoV-2 and serological response and both pre- and post-vaccination COVID-19, were collected in an anonymized database.

Anti-SARS-CoV-2 IgG antibody titers were obtained with the SARS-CoV-2 IgG II QUANT Alinity technique (Abbott^®^ (Abbott, Maidenhead, UK)) and defined the serological response to the vaccine. Humoral response to the vaccine was considered negative if <7.1 binding antibody units (BAU/mL) and positive if ≥7.1 BAU/mL. The first antibody titers available after complete vaccination were selected. Time elapsed (weeks) from the completion of the vaccination to the determination of the serological response was recorded, owing to its variability due to the non-availability of serological tests to measure vaccine humoral response at the initial phases of the vaccination period.

COVID-19 vaccination followed our local National Health System guidelines, and patients were administered one of the following four vaccines—(1) BNT162b2: two doses of 30 micrograms separated by 3 weeks; (2) mRNA-1273: two doses of 100 micrograms separated by 4 weeks; (3) AZD1222: two 0.5 milliliter doses separated by 10–12 weeks; (4) Ad.26.COV2.S: single dose of 0.5 milliliters. The type of vaccine and the number of doses administered were recorded. Additional boosters during the period of follow-up were also recorded.

### 2.4. Statistical Analysis

A descriptive analysis was performed with measures of central tendency (mean and/or median) and dispersion (standard deviation (SD) and/or interquartile range (IQR)) for the quantitative variables, showing frequencies and proportions for the qualitative variables. Analytical studies aimed at investigating the factors associated with the efficacy and/or safety of SARS-CoV-2 vaccine, documenting the variability in vaccination types, and considering their potential impact on both outcomes were carried out. The significant differences of the quantitative variables were assessed using the Student’s *t* test (two categories) or the ANOVA test (more than two categories) for normally distributed variables, while the Mann–Whitney test (two categories) or the Kruskal–Wallis test (more than two categories) were chosen for variables following a non-normal distribution. Chi-square or Fisher exact test were used for comparisons of qualitative variables.

Subsequently, multivariate logistic and linear regression analyses were performed, generating models fitted for factors that could influence immune response to vaccination, including as independent variables those that were significant in the bivariate analyses with the respective dependent variables. All the models were adjusted for age, sex, type of vaccine, number of doses administered, and time elapsed from vaccination to serological determination. For humoral response achievement, we used as a dependent variable the presence anti-SARS-CoV-2 IgG antibody titers ≥ 7.1 BAU/mL. In the case of the degree of response to vaccination, the values of the antibody titers were used as dependent variables. The final models were reached by means of a backward stepwise removal of variables with *p*-value > 0.15, except when that removal worsened the goodness of the model. To evaluate potential factors associated with vaccination adverse events and RA relapses, similar multivariable analyses were carried out. Values were considered statistically significant if *p* ≤ 0.05. Statistical analysis was performed with Stata v.14^®^ (StataCorp, College Station, TX, USA).

## 3. Results

### 3.1. Characteristics of the Study Population

Between December 2021 and April 2022, we analyzed 118 patients with RA who had received at least one dose of the SARS-CoV-2 vaccine. The mean (±SD) age of the study population was 65.5 ± 11.7 years (range: 36–91 years), with 87.3% being women, and 92.4% of European ancestry. Regarding age distribution, 35.6% of the patients were ≤60 years old, 28.0% were 60–70 years old, and 36.4% were older than 70 years. We did not find significant differences in the mean age or in the distribution of age groups between men and women (*p* = 0.204 and *p* = 0.288, respectively).

It is noteworthy that 35.6% were active smokers or ex-smokers, and 57.6% had comorbidities (Figure S1), the most frequent being dyslipidemia (41.5%) and high blood pressure (31.4%).

The main clinical-analytical characteristics of our population are shown in Table 1. The mean age of RA onset was 53.0 ± 13.6 years and extra-articular manifestations included rheumatoid nodules (6.8%), interstitial lung disease (6.0%), atlanto-axoid subluxation (1.7%), and keratoconjunctivitis sicca, secondary amyloidosis or osteoporosis (15.2%). Most of our patients (80.5%) had a low DAS28 score at the time of immunization, but 17.8% and 1.7% of them had moderate and high activity, respectively.

Prior RA treatment included GC 30.5%, methotrexate (MTX) (96.6%), hydroxychloroquine (22.0%), leflunomide (38.1%), and biologic DMARDS (bDMARDs) or JAK inhibitors (JAKinh) (49.1%). Regarding current therapies, most of the patients did not use GC treatment, and when required, it was prescribed at a low dose. The administration of conventional synthetic DMARDs (csDMARDs) was almost universal (94.1%), and MTX was the most frequently used drug (83.9%) followed by leflunomide (16.9%). Among bDMARDs (42.4%), TNF antagonists (aTNF) were the most frequently administered therapies (20.3%), followed by RTX (16.1%), with an average of 6.9 ± 4.0 treatment cycles. The use of csDMARDs as monotherapy (42.4%) was less common than in combination (51.7%), either of csDMARDs (9.3%) or csDMARDs with bDMARDs or JAKinh (42.4%), with an average number of drugs of 1.5 ± 0.6. Further details of current therapies can be seen in Table 2.

When assessing the immune response induced by vaccination, it is important to take into account that 17.8% of our population had previously been infected by SARS-CoV-2. The diagnosis was made by polymerase chain reaction (PCR) in 54.5% and by antigen test in 45.4%, either at primary care (68.2%) or hospital care (31.8%) settings. All cases were symptomatic with the most frequent manifestations being fever (66.7%) and cough (47.6%). COVID-19 was severe in six patients (5.1%) who required hospitalization, with an average stay lasting 3.9 ± 9.4 days. All of them required high-flow oxygen therapy, but none required intensive care unit (ICU) admission. Pre-vaccination infection was significantly associated with male sex (*p* = 0.016) in our population.

### 3.2. Vaccination against SARS-CoV-2

Among the patients, 95.8% received the complete vaccination schedule. All second doses of the vaccines were homologous. Five patients, who had previously tested positive for COVID-19, received just one dose of the vaccine (1 BNT162b2 and 4 AZD1222), following the initial vaccination recommendations of our Local Health Authorities. Another patient refused to receive the second dose of AZD1222 due to fear over the vaccine’s side effects.

**Figure 1:**
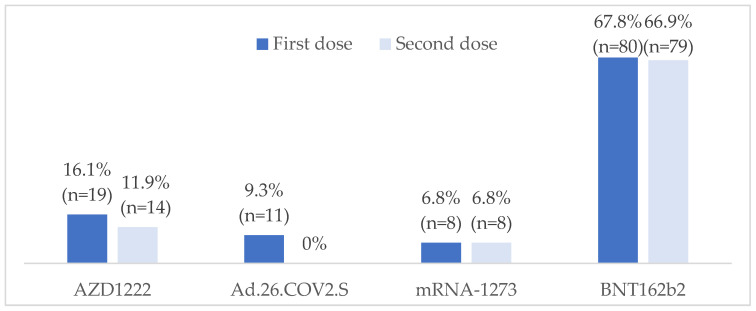
Types of COVID-19 vaccines administered. Additional doses of COVID-19 vaccine were administered to 104 (88.1%) patients, of autologous type in 57 cases, after a median time of 6.5 months (interquartile range: 4.1–12.4). BNT162b2 vaccine was the most frequently used (66.3%), followed by mRNA-1273 (33.7%).

Regarding the mechanism of action, mRNA vaccines (75.6%) predominated over adenovirus vector vaccines (25.4%). The most frequently administered vaccine was the BNT162b2 mRNA vaccine (67.8%), followed by the AZD1222 adenovirus vector vaccine (16.1%). The types of vaccines administered are shown in Figure 1.

### 3.3. Vaccine Efficacy

We analyzed the achievement of immunogenicity and the degree of humoral response as surrogate markers of vaccine effectiveness, and searched for the related factors. The mean (SD) time elapsed from the completion of the vaccination schedule to the measurement of anti-SARS-CoV-2 antibodies was 4.4 (2.2) months due to the non-availability of serological tests in the initial phases of the vaccination period. This difference helped us evaluate the persistence of response, as all the measurements were performed before additional vaccine boosters. Furthermore, we studied the potential impact of the serologic response on the incidence of post-vaccination COVID-19.

#### 3.3.1. Positive Serological Response and Degree of Response

The analysis of the overall population showed that 104 (88.1%) patients developed humoral immunogenicity after vaccination. Bivariate analyses were carried out to identify the factors associated with the achievement of serological response to the vaccine (Table 3).

In summary, the humoral response induced by the vaccine showed a significant inverse association with the smoking pack years (OR 0.96; 95% CI: 0.94–0.99; *p* < 0.001), extra-articular manifestations (OR 0.32; 95% CI: 0.09–1.26; *p* = 0.045), and anti-cyclic citrullinated peptide (aCCP) antibodies titer (OR 0.27; 95% CI: 0.05–1.12; *p* = 0.005), and was significantly associated with mRNA vaccines (OR 3.52; 95% CI: 1.12–11.07; *p* = 0.024).

We evaluated the impact of age by categorizing it into subgroups. Despite the fact that we found a numerically lower humoral response as age increased, the difference did not reach statistical significance. The same occurred with the number of doses of vaccine administered, as two doses rendered a higher percentage of vaccine humoral response than one dose. Time from vaccine administration to the measurement of serology was stratified in periods of 4 weeks. Interestingly, we found a high percentage of positive response to the vaccine in all subgroups, with a slight decrease from 8 weeks on (100% if ≤8 weeks vs. 80–87% when >8 weeks, *p* = 0.448). The persistence of response with mRNA vaccines was significantly higher than with adenovirus vector vaccines after 16 weeks from vaccine administration (91.3% vs. 60.0% positive serology, respectively; *p* = 0.002). 

As a 100% positive response to the vaccine was observed in patients that had been previously infected by SARS-CoV-2, we decided to stratify the analysis by previous COVID-19. Interestingly, all patients who had suffered previous COVID-19 achieved immunogenicity independently of age, type of vaccine and number of doses administered, and a period from vaccination to serology measurement. However, in patients without previous COVID-19, a non-significant decrease was observed with increasing age and time from vaccination to serology measurement (>8 weeks), and the patient who received one dose of vaccine did not achieve humoral response.

DMARDs were not significantly associated with achieving humoral immunogenicity, either in the global population or after stratifying by previous COVID-19 infection. However, peri-vaccine adjustments of DMARDs might have played a role since in at least 37.3% of the patients temporary MTX withdrawal for 1.5 ± 0.5 weeks was recorded. Regarding RTX, mean time from the last dose of RTX to vaccination was 12.0 ± 10.5 months (range: 0.5–36.7) and mean time to administration of further RTX cycles after vaccination was 5.2 ± 1.9 months (range: 1.6–8.2). No information was available for the rest of DMARDs in the electronic records.

**Figure 2:**
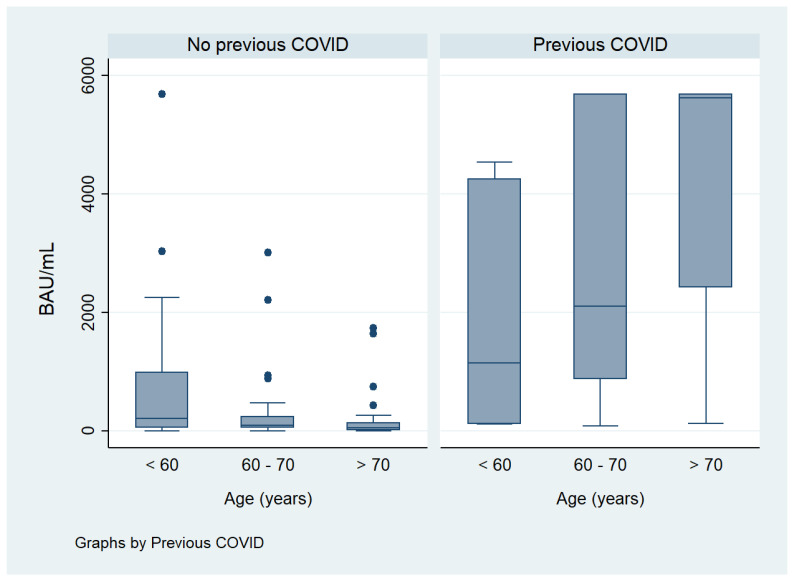
Influence of age on degree on humoral response stratified by previous COVID-19.

**Figure 3:**
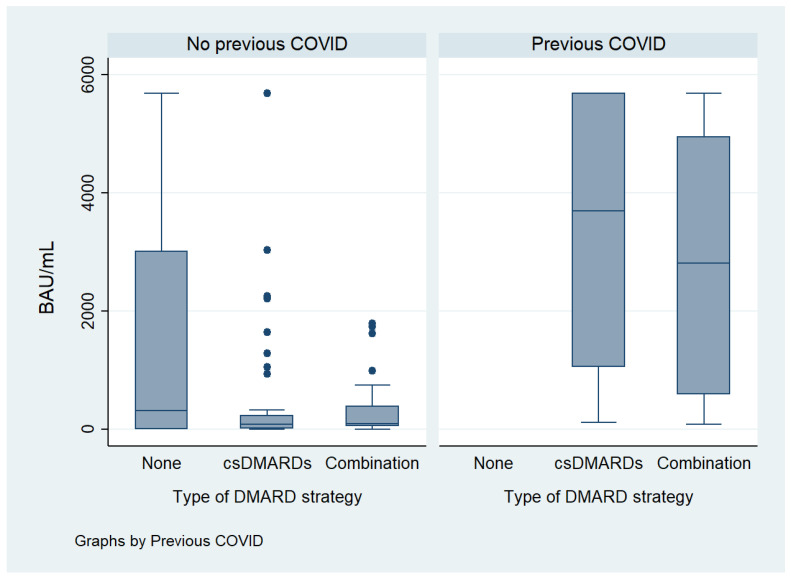
Influence of DMARD strategy on humoral response stratified by previous COVID-19. csDMARDs: conventional synthetic disease modifying anti-rheumatic drugs. It includes their use as monotherapy and a combination of different csDMARDs. Combination refers to the use of csDMARDs in association with biological DMARDs or JAK inhibitors.

**Figure 4:**
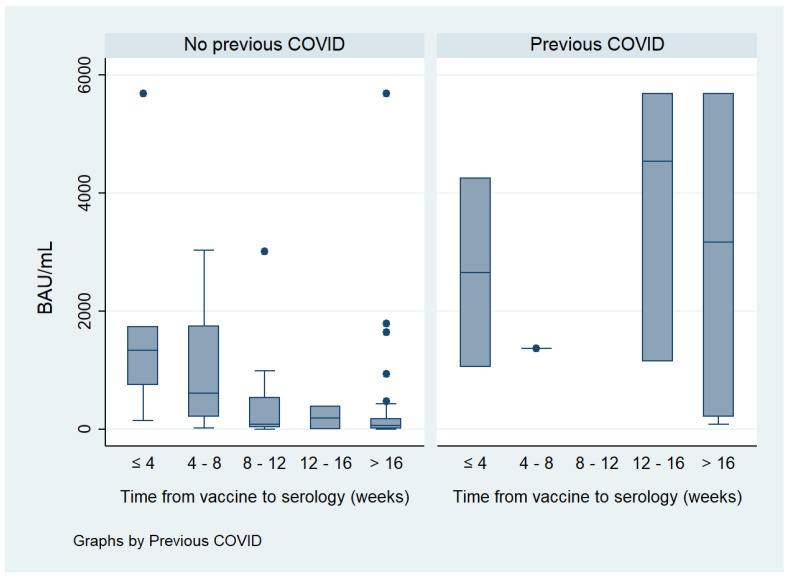
Persistence of humoral response stratified by previous COVID-19.

Regarding the degree of serological response, in bivariate analysis, it was significantly and directly associated with male sex (*p* = 0.019), CRP (*p* = 0.006) and previous COVID-19 infection (*p* < 0.001), and inversely related to ESR (*p* = 0.020). After stratifying by previous COVID-19, age (*p* = 0.030), csDMARDs use (*p* = 0.003) and time from vaccination to serology (*p* < 0.001) were significantly associated with lower humoral response (Figure 2, Figure 3 and Figure 4).

Stratification by the DMARD treatment strategy showed a lower degree of response for csDMARDs (in monotherapy or combination) with respect to the combination of csDMARDs with bDMARDs or JAKi (*p* = 0.018). Number of doses of vaccine and mRNA vaccines were significantly associated with a higher degree of response in patients with previous COVID-19 (*p* = 0.036 and *p* = 0.027, respectively). Numerically higher levels of antibodies were also found in patients without previous COVID-19, although it did not reach statistical significance.

Multivariate logistic and linear regression analysis were adjusted for age, sex, type of vaccine, number of doses administered, and time elapsed from vaccination to serological determination. Results in global population and in patients without previous COVID-19 infections are shown in Table 4 and Table 5.

#### 3.3.2. Post-Vaccination SARS-CoV-2 Infection

During follow-up, 22 (18.6%) patients suffered post-vaccination COVID-19. Median time from complete vaccination to infection was 30.4 weeks (interquartile range: 25–37). All of them were responders to the vaccine (BAUmL ≥ 7.1), and three of them had been previously infected. At the time of post-vaccination infection, 10 (45.4%) of them had already received a third booster dose. Of the three patients with previous COVID-19, two of them had received just one dose of the vaccine and the third one had been administered two doses and an additional booster dose. We did not find a significant association between post-vaccination infection and the number of vaccine doses received (*p* = 0.541) or degree of the serological response to the vaccine (*p* = 0.145). The majority of patients, 17 (77.3%), were symptomatic with a cough (40.9%), nasal congestion (36.4%), expectoration (27.3%), fever (27.3%) and malaise (27.3%) being the most frequent symptoms. Most notably, and in contrast to pre-vaccination infection, none of them required hospital admission. Bivariate analysis showed that post-vaccination SARS-CoV-2 infection was significantly associated with the presence of comorbidities such as deep vein thrombosis (*p* = 0.033) and asthma (*p* = 0.039), and with treatment with abatacept (ABA) (*p* = 0.033), identifying a trend towards association with younger age (*p* = 0.058) and a shorter RA evolution (*p* = 0.051). However, only the coexistence of asthma showed statistical significance in multivariate logistic regression analysis (OR 7.60, 95% CI 1.60–36.20, *p* = 0.011).

### 3.4. Vaccine Safety

**Figure 5:**
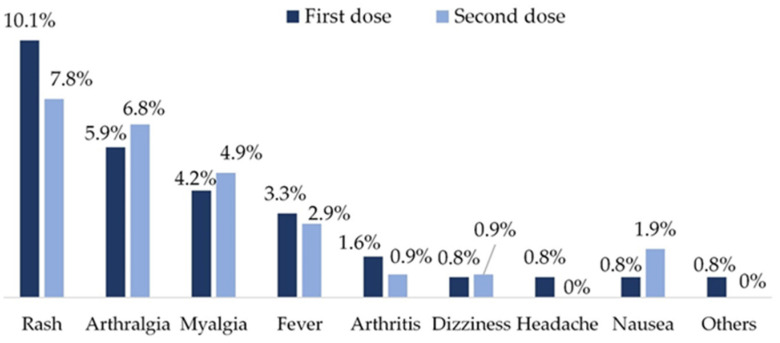
Adverse events of COVID-19 vaccines in our population.

Vaccination was generally well tolerated. AE following vaccination occurred in 19.5% (*n* = 23) of the patients after the first dose and in 18.6% (*n* = 19) after the second dose, but none of them were severe (Figure 5). All AE were considered by patients and/or their physicians to be mild or moderate in intensity and resolved in less than 7 days, generally spontaneously, or with a short course of acetaminophen or nonsteroidal anti-inflammatory drugs (NSAIDs). No patient required hospital admission or a readjustment of their background RA medication. The most frequent AE were skin rash and arthralgias.

Only two (1.7%) patients had mild reactivation of arthritis after vaccination: one of them after receiving both vaccine doses, and the other only after the first dose. These symptoms resolved without any readjustment of their RA treatment.

After the first dose of the vaccine, younger age (60.6 ± 13.7 versus 66.7 ± 10.9, *p* = 0.02) and receiving RTX treatment (8 [34.8%] versus 11 [11.6%], *p* = 0.007) were associated with the occurrence of AE. Only the RTX treatment maintained statistical significance in multivariate logistic regression analysis (OR 3.7, 95% CI 1.2–11.0, *p* = 0.020), with a trend towards significance for age (OR 0.96, 95% CI 0.9–1.0, *p* = 0.052). After the second dose of vaccine, in addition to younger age (60.4 ± 11.7 versus 67.3 ± 11.6, *p* = 0.020) and RTX treatment (6 [31.6%] versus 11 [13.1%], *p* = 0.050), mRNA-1273 vaccine (5 [26.3%] versus 4 [4.8%], *p* = 0.019) and having had AE after the first dose of COVID-19 vaccine (10 [52.6%] versus 10 [11.9%], *p* < 0.001) were also associated with the occurrence of AE. In multivariate logistic regression analysis, age (OR 0.95, 95% CI 0.91–0.99, *p* = 0.042) showed an inverse significant association with the occurrence of AE after the second dose of the COVID-19 vaccine, whereas a previous AE (OR 6.20, 95% CI 1.80–20.70, *p* = 0.052) showed a direct significant association with the occurrence of AE after the second dose. AEs were not related to either the serological response or its degree.

## 4. Discussion

Our data on the effectiveness and safety of COVID-19 vaccines in patients with RA are reassuring and support its use. The seroconversion rate of our RA patients was high (88.1%) and closer to the upper range described by other authors (61.8–94.0%) [21,22,23,24,25].

There are several possible explanations for these favorable humoral immunogenicity results in our population. Firstly, mRNA vaccines were the most frequently used in our population and showed a higher percentage of response achievement, higher levels of antibodies obtained, and the higher persistence of response after 16 weeks, in line with previous literature both in the general population and patients with RA [23,26,27,28,29]. Even boosters with heterologous mRNA vaccines in patients who had received initial treatment with adenoviral vector vaccines have been shown to improve seroconversion rates [30], and all our patients received a homologous vaccine strategy, with the predominance of mRNA vaccines. Our data did not allow us to evaluate the potential differences between different types of mRNA vaccines.

Furthermore, most of our patients received two doses of COVID-19 vaccines, and despite the fact that statistical significance was not reached, a numerically higher number of patients achieved immunogenicity after two doses of COVID-19 vaccine than after one dose (90.2% versus 75.0%, *p* = 0.097), in line with literature [31,32,33,34,35,36]. Even in patients previously infected with COVID-19, the degree of response was significantly higher after 2 doses of vaccine (*p* = 0.036), highlighting the importance of complete vaccination schedule in all patients.

### 4.1. Factors Involved in the Immunomodulatory Response to COVID-19 Vaccines

Additionally, at the time of vaccination, most of our patients were in remission or low disease activity and did not use GC (90%) or required low doses of prednisone (average 5 mg/d), which could be associated with the favorable humoral response to the vaccine, since some authors have reported a reduction in seroconversion rates in patients receiving prednisone at doses > 7.5 mg/d [21,22,27].

Previous SARS-CoV-2 infection was revealed to be one of the main factors associated with increased immunogenicity to COVID-19 vaccine in our population, in line with the data reported in the literature [23,31,34,37]. A study by Saleem et al. involving 100 patients with RA reported lower seroconversion rates (55.4%) in patients without previous COVID-19 than in those previously infected (100%) [31]. The equivalent rates in our patients were 85.6% and 100%, respectively. Additionally, we identified that previous COVID-19 was associated with a later decline in antibody levels to the vaccine (>16 weeks) compared with patients who had not suffered the infection (>4 weeks). This might help to better define the administration of booster doses.

The negative association of smoking with the humoral response found in our study is especially interesting given its known role in the pathogenesis of RA, the appearance of aCCP antibodies, and some of its comorbidities such as interstitial lung disease, and also its link with systemic inflammation in general. Smoking has also been associated with impairment of humoral response to infections and vaccines [38], but its impact on humoral response to COVID-19 vaccines has scarcely been investigated in the literature [39,40]. 

The negative association of aCCP antibodies with humoral immunogenicity is also of interest and might be connected with the immune dysfunction associated with RA, since these antibodies are a marker of poor prognosis in RA, and are related to more aggressive forms of the disease that require more intensive treatment. Despite the fact that we did not find a significant association between disease activity scores and response to the vaccine, in our population, patients with positive aCCP antibodies had significantly higher CDAI (5.0 versus 2.6, *p* = 0.048). Furthermore, alterations in B-cell activation and B-cell subpopulations described in RA, independently of clinical activity and DMARDs, might also play a role in response to vaccination. On the contrary, other studies have observed a significant association between the presence of aCCP antibodies and higher levels of specific anti-spike antibodies against SARS-CoV-2 [23,32]. Hence, further investigation is required.

Additionally, age was identified as a relevant factor inversely associated with seroconversion and the degree of humoral response, especially in people older than 70 years and without previous SARS-CoV-2 infection. Our data are in agreement with a phase 4 prospective study including 260 patients with RA and 104 healthy controls that observed lower seroconversion rates in older individuals (OR = 0.79, *p* < 0.001) [21]. Despite the existing discrepancies in the literature on the impact of age on the humoral response to the vaccine (seroconversion and/or neutralizing antibodies), studies that describe a lower seroconversion in older people [21,23,25,27,41,42] predominate over those that do not find age-related differences [24,34].

Relevant comorbidities, such as obesity, which has been associated with less effective humoral response to vaccines [17,24,43], did not seem to have a negative impact in our patients, probably due to its low prevalence (5.9%). On the other hand, discrepancies between studies on the effect of potential factors on the serological response to vaccines may be a reflection of the heterogeneity of the included RA patients.

### 4.2. Effect of DMARDs on the Response to COVID-19 Vaccination

Especially reassuring are the high seroconversion rates achieved in our patients who were mostly on DMARD treatment (94.1%), even in combination (51.7%). We did not find an association between the use of DMARD treatment and the achievement of immunogenicity, but their use was related to a lower degree of immunogenicity in patients without previous COVID-19. This aspect is of great interest and focusses on the importance of establishing DMARD readjustment strategies during vaccination.

The negative effect of MTX on the humoral response to the COVID-19 vaccine has been described for seroconversion, antibody titers, neutralizing antibodies or cellular response, even in the administration of booster doses, with a dose-dependent effect [21,22,24,31,44,45]. Recent investigations have demonstrated the benefits on immunogenicity of temporarily stopping MTX in the peri-vaccine period [28,46,47]. The fact that at least 37.8% of our patients stopped MTX for an average of 1.5 weeks after each COVID-19 vaccine, as shown in their electronic records, could be related to their immunogenicity achievement. It is also relevant that the average number of DMARDs used in combination in our patients was 1.5, since a greater reduction in seroconversion has been described with the greater number of DMARDs used [21,43].

Despite the fact that we have no statistical power to analyze specific DMARDs individually, patients undergoing treatment with non-MTX csDMARDs, aTNF, JAKinh, and IL6 receptor inhibitors had adequate humoral seroconversion, unless administered in combination, especially with MTX, is in line with literature [21,22,23,27,31,33,41,48]. Our ABA and RTX data should be interpreted with caution due to the small number of patients on these therapies. In the literature, ABA and especially RTX have been associated with a negative effect on the humoral response [21,22,23,27,31,33,41]. In fact, RTX has been described as the main predictor of a negative immunogenicity response, with the virtual abolition of antibody production [25,41,49]. However, it has been described that the probability of seroconversion increases with a lower cumulative dose of RTX and longer intervals between drug administration and vaccination [50,51], and in our series, both patients under RTX were on an optimization strategy. Their mean time from the last dose of RTX to vaccination was 12.0 ± 10.5 months (range: 0.5–36.7), and the mean time between vaccination and the administration of further RTX cycles was 5.2 ± 1.9 months (range: 1.6–8.2).

### 4.3. Effectiveness of COVID-19 Vaccines

Despite the importance of the ability of COVID-19 vaccines to generate an adequate protective antibody response in patients with RA, their effectiveness in reducing the severity or occurrence of new SARS-CoV-2 infections is probably more relevant. We consider this to be one of the main contributions of our study, since there are few data reported to date. Nearly 19% of our patients had a SARS-CoV-2 infection during the 6 months of follow-up. Notably, the majority of patients were either asymptomatic (17/22) or presented mild manifestations. No hospital admissions were required, unlike the pre-vaccine period in which 5% of patients with COVID-19 had suffered a severe infection.

Le Moine et al. found an incidence of COVID-19 of 8.9% six months after vaccination, and like us, without serious cases [33]. Cook et al. found a similar incidence of COVID-19 between mRNA vaccines (8.1% BNT162b2 versus 7.6% mRNA-1273) in patients with systemic autoimmune diseases [52]. Colmegna et al. found a self-reported incidence of SARS-CoV-2 infection of 14.5%, 12 months after vaccination [25], while Picchianti-Diamanti et al. described that 42% of the RA patients in their study presented with SARS-CoV-2 infection after an average time of 6 months since the last vaccine [53]. Although the majority were pauci-symptomatic, 8.5% required admission, with RTX being the main risk factor associated with admission [53]. In our study, the only factor associated with the incidence of COVID-19 was the presence of asthma.

In our population, 10 (45.4%) patients with post-vaccine infection had received a booster vaccine dose. This could be explained by the emergence of new SARS-CoV-2 strains and the decrease in antibody titers over time. In fact, a recent study evaluating the humoral immunity in 104 RA patients at 1, 3, and 6 months after receiving the full vaccination schedule concluded that it decreased significantly (*p* < 0.01) between 1 and 3 months, with an 8.9% incidence rate of post-vaccination infection, all cases being observed between 3 and 6 months after vaccination [33]. Furthermore, patients with RA have a greater risk of infection and hospitalization than the general population from 9 months after vaccination, reinforcing the importance of administering boosters and using other prevention strategies [54,55].

### 4.4. Safety of COVID-19 Vaccines

Adverse events of SARS-CoV-2 vaccination in our sample occurred in around 20% of the patients showed a similar profile to that described in the general population. Other authors have reported a similar frequency and severity of AEs in patients with autoimmune rheumatic diseases as found in healthy controls and other non-rheumatic autoimmune diseases reinforcing the safety of vaccines in patients with RA [56,57,58]. The percentage of AEs in our patients was generally lower than in literature (27.7–86%) and mostly mild [25,54,58,59,60,61]. Most authors also found that AEs were mostly mild to moderate, although 0.5%–4.2% serious AEs have been reported [56,61]. We found that AEs were associated with RTX treatment and younger age. Additionally, we identified an increased likelihood of suffering AE after the second vaccine dose in patients who had experienced AE after the first dose. Other studies have also observed higher frequency of AE at younger ages and in females [9,57,59,60]. In contrast to our data, which showed a similar percentage of AE after the first and second doses of the vaccine, the Korean College of Rheumatology argues that AE may be more intense after the second dose [9].

We found no differences in AE between vaccine types, although some authors have reported a higher frequency of AE with mRNA vaccines [26,56], while others have described a higher frequency of arthralgia with the AZD1222 vaccine [59].

Arthralgia was the second most frequent AE in our population after skin rash, in line with a study comparing the AEs of vaccination in 1198 RA patients and 1117 hospital workers that concluded that arthralgias were more frequent (3.1% vs. 0.8%, *p* < 0.0001) and longer lasting in RA patients, with no significant change in disease activity [62].

The low rate of RA flares (1.7%) in our patients could be related to the predominance of remission or low disease activity, in agreement with studies that found an association between flares after vaccination and disease activity [57,63]. Other authors have reported higher rates of RA flares (4.4–15.7%) that were mainly mild and self-limiting. However, some cases were moderate–severe and required changes in the background treatment (1.5–30%) [54,57,61,62,63,64,65]. We also found that RA flares were inversely related to age, in line with the study by Ma et al. [63].

### 4.5. Limitations

The main limitations of our study are the limited sample size, its retrospective design, and the lack of a control group. Well-controlled disease activity of most of our patients may have contributed to the favorable response to COVID-19 vaccines in our population, in terms of effectiveness and safety, and may not be extrapolated to other groups of patients with more active disease. The retrospective design of our study with the data obtained from the electronic records of the patients does not allow us to exclude the possibility that peri-vaccine adjustment of DMARDs could have been higher and had an effect on the achievement of an adequate humoral response to the vaccine. Additionally, we could not thoroughly investigate the effect of specific DMARDs on the response to the vaccine due to the low representation of various therapies, so the discussed inferences should be interpreted with caution. Due to the lack of the availability of the serological test at the beginning of the vaccination period, measurements were obtained at different time intervals after vaccination. In order to mitigate its impact on the study, multivariate analyses were adjusted for this factor. However, this fact has allowed us to have information on the persistence of vaccine response in real-world conditions, obtaining relevant information to help define booster strategies. In any case, if the determinations had been closer in time to the administration of the vaccine, it is expected that the seroconversion rates would have been even greater, thus reinforcing the results obtained. Unfortunately, we do not have data on the serological status of our patients prior to vaccination since our health authorities did not recommend its performance at that moment. Additionally, we cannot completely exclude that some patients had suffered a subclinical SARS-CoV-2 infection prior to vaccination due to the recommendation to perform screening tests only in patients with suspected symptoms.

## 5. Conclusions

Humoral immunogenicity induced by SARS-CoV-2 vaccines can be considered reasonably adequate in our real-world study that includes RA patients with favorable disease control. The factors most strongly associated with a greater humoral response to vaccines were having had previous SARS-CoV-2 infection and receiving mRNA vaccines, while age and csDMARD therapeutic strategies seemed to be inversely related with the degree of immunogenicity. Additionally, the persistence of vaccine response was longer in patients previously infected by SARS-CoV-2, a fact that may help define future vaccination strategies.

Despite the fact that the incidence of COVID-19 infection after vaccination remained high, a reduction in its severity was noted. No hospital admissions were required, thus reinforcing the effectiveness of these vaccines. The good safety profile and acceptable tolerability of vaccines in our population of patients with RA should also be highlighted. Further prospective studies with a higher sample size are needed to determine the evolution of antibody titers over time and the number and frequency of doses required to achieve better protection against SARS-CoV-2 in patients with RA.

## Figures and Tables

**Table 1:** Clinical-analytical characteristics of the study population.

Variables	Descriptive Analysis
**Sociodemographic features**	
Age (years)	65.5 ± 11.7
Sex (female)	103 (87.3)
Smoking *	42 (35.6)
Pack years #	20.5 (10.5–120.0)
Comorbidities	68 (57.6)
**Rheumatoid arthritis characteristics**	
RA evolution (years)	10.1 (4.7–19.1)
RA onset (years)	53.0 ± 13.6
Rheumatoid factor (positive)	79 (66.9)
Titer (IU/mL)	42 (17–130)
aCCP antibodies (positive)	63 (53.4)
Titer (IU/mL)	375 (238–460)
Erosive RA	32 (27.1)
Extra-articular manifestations	26 (22.0)
ESR (mm/h)	14 (6–26)
CRP (mg/dL)	0.5 ± 0.8
HAQ	0.250 (0–0.875)
DAS28	2.4 ± 1.1

aCCP antibodies: anti-cyclic citrullinated peptide antibodies; CRP: C-reactive protein; DAS28: Disease Activity Score of 28 joints; dL: deciliter; ESR: erythrocyte sedimentation rate; h: hour; HAQ: Health Assessment Questionnaire; IU/mL: international units per milliliter; mg: milligram; RA: rheumatoid arthritis. Numerical variables with normal distribution are expressed as mean ± standard deviation and non-normal variables as median (interquartile range), while qualitative variables are expressed as number (percentage). * Smoking includes current and prior smokers. # Pack years refers only to patients with current or prior smoking habits.

**Table 2:** Current rheumatoid arthritis treatments.

RA Treatment	Descriptive Analysis
**Glucocorticoids**	11 (9.3)
Dose (mg/day)	5.3 ± 2.2
**Conventional synthetic DMARDs**	
Methotrexate	99 (83.9)
Dose (mg/week)	13.2 ± 4.5
Leflunomide	20 (16.9)
Dose (mg/day) *	20 (10–20)
Hydroxycloroquine	6 (5.1)
Sulfasalazine	3 (2.5)
**Biological DMARDs**	
**Abatacept** (sc)	2 (1.7)
**Rituximab** (iv)	19 (16.1)
**IL-6 Receptor inhibitors**	
Tocilizumab (sc or iv)	2 (1.7)
Sarilumab (sc)	3 (2.5)
**TNFα inhibitors**	
Etanercept	14 (11.9)
Adalimumab	4 (3.4)
Certolizumab	4 (3.4)
Golimumab	1 (0.8)
Infliximab (iv)	1 (0.8)
**JAK inhibitors**	
Tofacitinib	2 (1.7)
Baricitinib	3 (2.5)

DMARDs: disease modifying anti-rheumatic drugs; IL-6: interleukin-6; iv: intravenous; JAK: Janus Kinase; mg: milligram; RA: rheumatoid arthritis; sc: subcutaneous; TNFα: tumor necrosis factor alpha. Numerical variables are expressed as mean ± standard deviation and qualitative variables as number (percentage). * Leflunomide dose is expressed as median (interquartile range).

**Table 3:** Factors associated with serological response to the COVID-19 vaccine in bivariate analysis.

	Serological Response			
Variables	Yes	No	*p*	OR (95% CI)
Age (years)	65.2 ± 11.7	67.1 ± 12.1	0.233	-
<60	38 (90.5)	4 (9.5)	0.882	-
60–70	29 (87.9)	4 (12.1)		
>70	37 (86.0)	6 (13.9)		
Sex (female)	92 (88.5)	11 (78.6)	0.385	-
Smoking (pack years)	6.9 ± 15.2	23.0 ± 31.8	**<0.001**	**0.96 (0.94–0.99)**
Comorbidities	58 (55.8)	10 (71.4)	0.389	-
RA evolution	11.6 ± 8.6	15.9 ± 15.3	0.994	-
RA onset	53.5 ± 13.1	49.6 ± 17.6	0.319	-
Rheumatoid factor (positive)	72 (69.2)	7 (50.0)	0.151	-
Titer (IU/mL)	117.6 ± 282.1	66.1 ± 137.0	0.999	
aCCP antibodies (positive)	52 (82.5)	52 (94.5)	**0.056**	**0.27 (0.07–1.03)**
Titer (IU/mL)	196.3 ± 252.5	240.1 ± 285.4	**0.005**	
Erosive RA	26 (25.0)	6 (42.9)	0.158	-
Extra-articular manifestations	20 (19.2)	6 (42.9)	**0.045**	**0.32 (0.10–1.02)**
ESR (mm/h)	20.0 ± 19.0	19.0 ± 15.9	0.855	-
CRP (mg/dL)	0.5 ± 0.6	0.8 ± 1.4	0.239	-
HAQ	0.6 ± 0.7	0.4 ± 0.6	0.810	-
DAS28	2.4 ± 1.1	2.5 ± 0.6	0.110	-
Previous COVID-19	22 (100)	0 (0)	**0.069**	**1**
Glucocorticoids	8 (7.7)	3 (21.4)	0.124	-
csDMARDs	99 (95.2)	12 (85.7)	0.194	-
bDMARDs	46 (44.2)	4 (28.6)	0.389	-
JAK inhibitors	5 (4.8)	0 (0)	1.000	-
Type of vaccine				
Adenovirus vector	23 (76.7)	7 (23.3)	**0.024**	**3.52 (1.12–11.07)**
mRNA	81 (92.0)	7 (7.9)		
Number of vaccine doses				
1 dose	5 (83.3)	1 (16.7)	0.487	-
2 doses	91 (90.1)	10 (9.9)		

aCCP: anti-cyclic citrullinated peptide antibodies; bDMARDs: biological disease modifying anti-rheumatic drugs; COVID-19: coronavirus disease 2019; CRP: C-reactive protein; csDMARDs: conventional synthetic disease modifying anti-rheumatic drugs; DAS28: Disease Activity Score of 28 joints with ESR; d: day; dL: deciliter; ESR: erythrocyte sedimentation rate; g: gram; h: hour; HAQ: Health Assessment Questionnaire; IU/mL: international units per milliliter; iv: intravenous; mg: milligram; mRNA: messenger ribonucleic acid; RA: rheumatoid arthritis; sc: subcutaneous; w: week. Numerical variables are expressed as mean ± standard deviation and qualitative variables as number (percentage). Factors with a statistically significant association with serological response or close to statistical significance are shown in bold.

**Table 4:** Factors associated with the achievement of immune response to vaccine (multivariate analysis).

Response to Vaccine	Multivariate Analysis		
Variables	OR	95% CI	*p*
**Global Population**			
Smoking (pack years)	0.93	0.89–0.98	0.005
aCCP antibodies (positive)	0.10	0.01–0.79	0.030
Type of COVID-19 vaccine			
Adenovirus vector	Reference		
mRNA	36.62	1.96–684.0	0.016
**Without Previous COVID-19**			
Smoking (pack years)	0.93	0.89–0.99	0.019
aCCP antibodies (positive)	0.05	0.01–0.64	0.021
Type of COVID-19 vaccine			
Adenovirus vector	Reference		
mRNA	36.62	1.12–1548.72	0.043

aCCP: anti-cyclic citrullinated peptide antibodies; CI: confidence interval; COVID-19: coronavirus disease 2019; mRNA: messenger ribonucleic acid; OR: odds ratio.

**Table 5:** Factors associated with the degree of immune response to vaccine (multivariate analysis).

Degree of Response to Vaccine	Multivariate Analysis		
Variables	Coefficient	95% CI	*p*
**Global Population**			
**Time from Vaccination to Serology**			
≤4 weeks	Reference		
4–8 weeks	−365.6	−1555.5, 824.2	0.543
8–12 weeks	−217.5	−1581.2, 1146.1	0.752
12–16 weeks	−1215.5	−2834.9, 403.9	0.140
>16 weeks	−1370.4	−2387.8, −353.0	0.009
**Type of COVID-19 Vaccine**			
Adenovirus vector	Reference		
mRNA	1381.3	451.3, 2311.2	0.004
**csDMARD**			
Monotherary or csDMARD combination	−1459.8	−2562.7, −356.9	0.010
Combined with bDMARD or JAKi	−1790.9	−2897.4, −684.4	0.002
**Previous SARS-CoV-2 Infection**	3122.3	2363.1, 3881.4	<0.001
**Without Previous COVID-19**			
**Time from Vaccination to Serology**			
≤4 weeks	Reference		
4–8 weeks	−826.7	−1648.8, −4.5	0.049
8–12 weeks	−1007.2	−1928.3, −86.1	0.032
12–16 weeks	−1306.8	−2664.5, 50.8	0.059
>16 weeks	−1551.7	−2265.2, −838.1	<0.001
**Age Groups (Years Old)**			
<60	Reference		
60–70	−186.3	−650.4, 277.7	0.427
>70	−423.8	−859.6, 12.0	0.057
**Type of COVID-19 Vaccine**			
Adenovirus vector	Reference		
mRNA	659.5	142.7, 1176.4	0.013
**csDMARD**			
Monotherary or csDMARD combination	−1203.353	−1893.1, −513.6	0.001
Combined with bDMARD or JAKi	−1454.7	−2139.9, −769.5	<0.001

bDMARDs: biological disease modifying anti-rheumatic drugs; CI: confidence interval; COVID-19: coronavirus disease 2019; csDMARDs: conventional synthetic disease modifying anti-rheumatic drugs; JAKi: Janus kinase inhibitors; mRNA: messenger ribonucleic acid.

## Data Availability

All data relevant to the study are included in the article. Additional data are available upon reasonable request to the corresponding authors.

## Supplementary Materials

The following supporting information can be downloaded at: https://www.mdpi.com/article/10.3390/vaccines12060672/s1, Figure S1: Comorbidities of the RA population; Table S1: Description of analyzed variables.

## References

[1] Seoane-Mato D., Sánchez-Piedra C., Díaz-González F., Bustabad S. (2018). Prevalence of rheumatic diseases in adult population in Spain. EPISER 2016 study. Ann. Rheum. Dis..

[2] García de Yébenes M.J., Loza E. (2018). Rheumatoid arthritis: Epidemiology and socio-health impact. Clin. Rheumatol. Suppl..

[3] Smolen J.S., Landewé R.B.M., Bergstra S.A., Kerschbaumer A., Sepriano A., Aletaha D., Caporali R., Edwards C.J., Hyrich K.L., Pope J.E. (2023). EULAR recommendations for the management of rheumatoid arthritis with synthetic and biological disease-modifying antirheumatic drugs: 2022 update. Ann. Rheum. Dis..

[4] Brito-Zerón P., Sisó-Almirall A., Flores-Chavez A., Retamozo S., Ramos-Casals M. (2021). SARS-CoV-2 infection in patients with systemic autoimmune diseases. Clin. Exp. Rheumatol..

[5] Williamson E.J., Walker A.J., Bhaskaran K., Bacon S., Bates C., Morton C.E., Curtis H.J., Mehrkar A., Evans D., Inglesby P. (2020). Factors associated with COVID-19-related death using OpenSAFELY. Nature.

[6] Hasseli R., Mueller-Ladner U., Hoyer B.F., Krause A., Lorenz H.-M., Pfeil A., Richter J., Schäfer M., Schmeiser T., Strangfeld A. (2021). Older age, comorbidity, glucocorticoid use and disease activity are risk factors for COVID-19 hospitalisation in patients with inflammatory rheumatic and musculoskeletal diseases. RMD Open.

[7] Schulze-Koops H., Krueger K., Vallbracht I., Hasseli R., Skapenko A. (2021). Increased risk for severe COVID-19 in patients with inflammatory rheumatic diseases treated with rituximab. Ann. Rheum. Dis..

[8] Curtis J.R., Johnson S.R., Anthony D.D., Arasaratnam R.J., Baden L.R., Bass A.R., Calabrese C., Gravallese E.M., Harpaz R., Kroger A. (2021). American College of Rheumatology Guidance for COVID-19 Vaccination in Patients with Rheumatic and Musculoskeletal Diseases: Version 3. Arthritis Rheumatol..

[9] Park J.K., Lee E.B., Shin K., Sung Y.-K., Kim T.H., Kwon S.-R., Lee M.S., Hong S.-J., Choi B.Y., Lee S.-S. (2021). COVID-19 Vaccination in Patients with Autoimmune Inflammatory Rheumatic Diseases: Clinical Guidance of the Korean College of Rheumatology. J. Korean Med. Sci..

[10] Alten R., Bingham C.O., Cohen S.B., Curtis J.R., Kelly S., Wong D., Genovese M.C. (2016). Antibody response to pneumococcal and influenza vaccination in patients with rheumatoid arthritis receiving abatacept. BMC Musculoskelet. Disord..

[11] Kapetanovic M.C., Saxne T., Jönsson G., Truedsson L., Geborek P. (2013). Rituximab and abatacept but not tocilizumab impair antibody response to pneumococcal conjugate vaccine in patients with rheumatoid arthritis. Arthritis Res. Ther..

[12] Furer V., Rondaan C., Heijstek M.W., Agmon-Levin N., Van Assen S., Bijl M., Breedveld F.C., D’Amelio R., Dougados M., Kapetanovic M.C. (2020). 2019 update of EULAR recommendations for vaccination in adult patients with autoimmune inflammatory rheumatic diseases. Ann. Rheum. Dis..

[13] Hua C., Barnetche T., Combe B., Morel J. (2014). Effect of methotrexate, anti-tumor necrosis factor α, and rituximab on the immune response to influenza and pneumococcal vaccines in patients with rheumatoid arthritis: A systematic review and meta-analysis. Arthritis Care Res..

[14] Park J.K., Lee Y.J., Shin K., Ha Y.-J., Lee E.Y., Song Y.W., Choi Y., Winthrop K.L., Lee E.B. (2018). Impact of temporary methotrexate discontinuation for 2 weeks on immunogenicity of seasonal influenza vaccination in patients with rheumatoid arthritis: A randomised clinical trial. Ann. Rheum. Dis..

[15] Curtis J.R., Johnson S.R., Anthony D.D., Arasaratnam R.J., Baden L.R., Bass A.R., Calabrese C., Gravallese E.M., Harpaz R., Kroger A. (2023). American College of Rheumatology Guidance for COVID-19 Vaccination in Patients with Rheumatic and Musculoskeletal Diseases: Version 5. Arthritis Rheumatol..

[16] Grupo de Trabajo Técnico de Vacunación COVID-19, de la Ponencia de Programa y Registro de Vacunaciones Estrategia de Vacunación Frente a COVID-19 en España: Actualización 3. Ministerio de Sanidad. https://www.sanidad.gob.es/profesionales/saludPublica/prevPromocion/vacunaciones/covid19/Actualizaciones_Estrategia_Vacunacion/docs/COVID-19_Actualizacion3_EstrategiaVacunacion.pdf.

[17] Falahi S., Kenarkoohi A. (2022). Host factors and vaccine efficacy: Implications for COVID-19 vaccines. J. Med. Virol..

[18] Rondaan C., Furer V., Heijstek M.W., Agmon-Levin N., Bijl M., Breedveld F.C., D’amelio R., Dougados M., Kapetanovic M.C., Van Laar J.M. (2019). Efficacy, immunogenicity and safety of vaccination in adult patients with autoimmune inflammatory rheumatic diseases: A systematic literature review for the 2019 update of EULAR recommendations. RMD Open..

[19] Arnett F.C., Edworthy S.M., Bloch D.A., Mcshane D.J., Fries J.F., Cooper N.S., Healey L.A., Kaplan S.R., Liang M.H., Luthra H.S. (1988). The American Rheumatism Association 1987 revised criteria for the classification of rheumatoid arthritis. Arthritis Rheum..

[20] Aletaha D., Neogi T., Silman A.J., Funovits J., Felson D.T., Bingham C.O., Birnbaum N.S., Burmester G.R., Bykerk V.P., Cohen M.D. (2010). 2010 Rheumatoid arthritis classification criteria: An American College of Rheumatology/European League Against Rheumatism collaborative initiative. Ann. Rheum. Dis..

[21] Medeiros-Ribeiro A.C., Bonfiglioli K.R., Domiciano D.S., Shimabuco A.Y., da Silva H.C., Saad C.G.S., Yuki E.F.N., Pasoto S.G., Araujo C.S.R., Nakai T.L. (2022). Distinct impact of DMARD combination and monotherapy in immunogenicity of an inactivated SARS-CoV-2 vaccine in rheumatoid arthritis. Ann. Rheum. Dis..

[22] Jena A., Mishra S., Deepak P., Kumar M.P., Sharma A., Patel Y.I., Kennedy N.A., Kim A.H., Sharma V., Sebastian S. (2022). Response to SARS-CoV-2 vaccination in immune mediated inflammatory diseases: Systematic review and meta-analysis. Autoimmun. Rev..

[23] Nemeth D., Vago H., Tothfalusi L., Ulakcsai Z., Becker D., Szabo Z., Rojkovich B., Merkely B., Nagy G. (2022). Factors influencing the SARS-CoV-2 infection and vaccination induced immune response in rheumatoid arthritis. Front. Immunol..

[24] Ammitzbøll C., Thomsen M.K., Andersen J.B., Bartels L.E., Hermansen M.-L.F., Johannsen A.D., Mistegaard C.E., Mikkelsen S., Vils S.R., Erikstrup C. (2023). COVID-19 vaccination in patients with rheumatic diseases leads to a high seroconversion rate and reduced self-imposed isolation and shielding behaviour. Mod. Rheumatol..

[25] Colmegna I., Valerio V., Amiable N., Useche M., Rampakakis E., Flamand L., Rollet-Labelle E., Bessette L., Fitzcharles M.-A., Hazel E. (2023). COVID-19 Vaccine in Immunosuppressed Adults with Autoimmune rheumatic Diseases (COVIAAD): Safety, immunogenicity and antibody persistence at 12 months following Moderna Spikevax primary series. RMD Open.

[26] Jiesisibieke Z.L., Liu W.Y., Yang Y.P., Chien C.-W., Tung T.-H. (2023). Effectiveness and Safety of COVID-19 Vaccinations: An Umbrella Meta-Analysis. Int. J. Public Health.

[27] Le Moine C., Soyfoo M.S., Mekkaoui L., Dahma H., Tant L. (2022). Impaired Humoral Immunogenicity of SARS-CoV-2 Vaccination in Patients with Rheumatoid Arthritis. J. Rheumatol..

[28] Martínez-Fleta P., Vicente-Rabaneda E.F., Triguero-Martínez A., Roy-Vallejo E., Uriarte-Ecenarro M., Gutiérrez-Rodríguez F., Quiroga-Colina P., Romero-Robles A., Montes N., García-Castañeda N. (2024). Beneficial effect of temporary methotrexate interruption on B and T cell responses upon SARS-CoV-2 vaccination in patients with rheumatoid arthritis or psoriatic arthritis. NPJ Vaccines.

[29] Romero-Ibarguengoitia M.E., Rivera-Salinas D., Sarti R., Levi R., Mollura M., Garza-Silva A., Rivera-Cavazos A., Hernández-Ruíz Y.G., Barco-Flores I.A., González-Cantú A. (2023). Efficacy of Six Different SARS-CoV-2 Vaccines during a Six-Month Follow-Up and Five COVID-19 Waves in Brazil and Mexico. Vaccines.

[30] Hitchon C.A., Mesa C., Bernstein C.N., Marrie R.A., Card C., O’brien S.F., Kim J. (2023). Immunogenicity and safety of mixed COVID-19 vaccine regimens in patients with immune-mediated inflammatory diseases: A single-centre prospective cohort study. BMJ Open.

[31] Saleem B., Ross R.L., Bissell L.A., Aslam A., Mankia K., Duquenne L., Corsadden D., Carter C., Hughes P., Nadat F.A. (2022). Effectiveness of SARS-CoV-2 vaccination in patients with rheumatoid arthritis (RA) on DMARDs: As determined by antibody and T cell responses. RMD Open.

[32] Limoges M.A., Lortie A., Demontier É., Quenum A.J.I., Lessard F., Drouin Z., Carrier N., Nguimbus L.M., Beaulieu M.-C., Boire G. (2023). SARS-CoV-2 mRNA vaccine-induced immune responses in rheumatoid arthritis. J. Leuk. Biol..

[33] Le Moine C., Soyfoo M.S., Mekkaoui L., Dahma H., Tant L. (2023). Waning humoral immunity of SARS-CoV-2 vaccination in a rheumatoid arthritis cohort and the benefits of a vaccine booster dose. Clin. Exp. Rheumatol..

[34] Benoit J.M., Breznik J.A., Ang J.C., Bhakta H., Huynh A., Cowbrough B., Baker B., Heessels L., Lodhi S., Yan E. (2023). Immunomodulatory drugs have divergent effects on humoral and cellular immune responses to SARS-CoV-2 vaccination in people living with rheumatoid arthritis. Sci. Rep..

[35] Ammitzbøll C., Thomsen M.K., Andersen J.B., Jensen J.M.B., Bayarri-Olmos R., Garred P., Hermansen M.-L.F., Johannsen A.D., Larsen M.L., Mistegaard C.E. (2024). Revaccination of patients with systemic lupus erythematosus or rheumatoid arthritis without an initial COVID-19 vaccine response elicits seroconversion in half of the patients. Clin. Exp. Rheumatol..

[36] Cheung M.W., Dayam R.M., Shapiro J.R., Law J.C., Chao G.Y.C., Pereira D., Goetgebuer R.L., Croitoru D., Stempak J.M., Acheampong L. (2023). Third and Fourth Vaccine Doses Broaden and Prolong Immunity to SARS-CoV-2 in Adult Patients with Immune-Mediated Inflammatory Diseases. J. Immunol..

[37] Zamani B., Moradi Hasan-Abad A., Piroozmand A., Dehghani M., Arfaatabar M., Motedayyen H. (2023). Immunogenicity and safety of the BBIBP-CorV vaccine in patients with autoimmune inflammatory rheumatic diseases undergoing immunosuppressive therapy in a monocentric cohort. Immun. Inflamm. Dis..

[38] Qiu F., Liang C.L., Liu H., Zeng Y.-Q., Hou S., Huang S., Lai X., Dai Z. (2017). Impacts of cigarette smoking on immune responsiveness: Up and down or upside down?. Oncotarget.

[39] Nomura Y., Sawahata M., Nakamura Y., Kurihara M., Koike R., Katsube O., Hagiwara K., Niho S., Masuda N., Tanaka T. (2021). Age and Smoking Predict Antibody Titres at 3 Months after the Second Dose of the BNT162b2 COVID-19 Vaccine. Vaccines.

[40] Costa C., Migliore E., Galassi C., Scozzari G., Ciccone G., Coggiola M., Pira E., Scarmozzino A., La Valle G., Cassoni P. (2022). Factors Influencing Level and Persistence of Anti SARS-CoV-2 IgG after BNT162b2 Vaccine: Evidence from a Large Cohort of Healthcare Workers. Vaccines.

[41] Frommert L.M., Arumahandi de Silva A.N., Zernicke J., Scholz V., Braun T., Jeworowski L.M., Schwarz T., Tober-Lau P., Hagen A.T., Habermann E. (2022). Type of vaccine and immunosuppressive therapy but not diagnosis critically influence antibody response after COVID-19 vaccination in patients with rheumatic disease. RMD Open.

[42] Dudley H.M., O’Mara M., Auma A., Gong J., Ross Y., Gurevich N., Carbone S., Reihs A., Nguyen Y., McComsey G.A. (2023). Rheumatoid arthritis and older age are associated with lower humoral and cellular immune response to primary series COVID-19 mRNA vaccine. Vaccine.

[43] Eerike M., Parimi V.P., Pyati A., Sundaramurthy R., Sakthivadivel V., Pidugu A.B., Surapareddy B., Ramineni N.T., Priyadarshini R., Patil P.P. (2024). Clinical and immunological responses to COVID-19 vaccination in rheumatoid arthritis patients on disease modifying antirheumatic drugs: A cross-sectional study. J. Rheum. Dis..

[44] Shirata M., Ito I., Tanaka M., Murata K., Murakami K., Ikeda H., Oi I., Hamao N., Nishioka K., Hayashi Y. (2023). Impact of methotrexate on humoral and cellular immune responses to SARS-CoV-2 mRNA vaccine in patients with rheumatoid arthritis. Clin. Exp. Med..

[45] Tedeschi S.K., Solomon D.H., Chen Y., Ellrodt J., Whelan M.G., Stratton J., Hayashi K., Whiteman N.B., Chen L., Adejoorin I. (2023). Humoral and cellular immune responses in persons with rheumatoid arthritis after a third dose of mRNA COVID-19 vaccine. Semin. Arthritis Rheum..

[46] Araujo C.S.R., Medeiros-Ribeiro A.C., Saad C.G.S., Bonfiglioli K.R., Domiciano D.S., Shimabuco A.Y., Silva M.S.R., Yuki E.F.N., Pasoto S.G., Pedrosa T. (2022). Two-week methotrexate discontinuation in patients with rheumatoid arthritis vaccinated with inactivated SARS-CoV-2 vaccine: A randomised clinical trial. Ann. Rheum. Dis..

[47] Habermann E., Gieselmann L., Tober-Lau P., Klotsche J., Albach F.N., Hagen A.T., Zernicke J., Ahmadov E., de Silva A.N.A., Frommert L.M. (2022). Pausing methotrexate prevents impairment of Omicron BA.1 and BA.2 neutralisation after COVID-19 booster vaccination. RMD Open.

[48] Serra López-Matencio J.M., Vicente-Rabaneda E.F., Alañón E., Oyarzabal A.A., Fleta P.M., Castañeda S. (2023). COVID-19 Vaccination and Immunosuppressive Therapy in Immune-Mediated Inflammatory Diseases. Vaccines.

[49] Md Yusof M.Y., Arnold J., Saleem B., Vandevelde C., Dass S., Savic S., Vital E.M., Emery P. (2023). Breakthrough SARS-CoV-2 infections and prediction of moderate-to-severe outcomes during rituximab therapy in patients with rheumatic and musculoskeletal diseases in the UK: A single-centre cohort study. Lancet Rheumatol..

[50] Furer V., Eviatar T., Zisman D., Peleg H., Braun-Moscovici Y., Balbir-Gurman A., Paran D., Levartovsky D., Zisapel M., Elalouf O. (2022). Predictors of Immunogenic Response to the BNT162b2 mRNA COVID-19 Vaccination in Patients with Autoimmune Inflammatory Rheumatic Diseases Treated with Rituximab. Vaccines.

[51] van der Togt C.J.T., Ten Cate D.F., den Broeder N., Rahamat-Langendoen J., Bemt B.J.F.v.D., Broeder A.A.D. (2022). Humoral response to coronavirus disease-19 vaccines is dependent on dosage and timing of rituximab in patients with rheumatoid arthritis. Rheumatology.

[52] Cook C.E., Patel N.J., Fu X., Wang X., Kawano Y., Vanni K.M., Qian G., Banasiak E., Kowalski E., Choi H.K. (2023). Comparative Effectiveness of BNT162b2 and mRNA-1273 Vaccines Against COVID-19 Infection Among Patients with Systemic Autoimmune Rheumatic Diseases on Immunomodulatory Medications. J. Rheumatol..

[53] Picchianti-Diamanti A., Navarra A., Aiello A., Laganà B., Cuzzi G., Salmi A., Vanini V., Maggi F., Meschi S., Matusali G. (2023). Older Age, a High Titre of Neutralising Antibodies and Therapy with Conventional DMARDs Are Associated with Protection from Breakthrough Infection in Rheumatoid Arthritis Patients after the Booster Dose of Anti-SARS-CoV-2 Vaccine. Vaccines.

[54] Picchianti Diamanti A., Navarra A., Cuzzi G., Aiello A., Salemi S., Di Rosa R., De Lorenzo C., Vio D., Sebastiani G., Ferraioli M. (2023). The Third Dose of BNT162b2 COVID-19 Vaccine Does Not “Boost” Disease Flares and Adverse Events in Patients with Rheumatoid Arthritis. Biomedicines.

[55] Li H., Wallace Z.S., Sparks J.A., Lu N., Wei J., Xie D., Wang Y., Zeng C., Lei G., Zhang Y. (2023). Risk of COVID-19 Among Unvaccinated and Vaccinated Patients with Rheumatoid Arthritis: A General Population Study. Arthritis Care Res..

[56] Naveen R., Parodis I., Joshi M., Sen P., Lindblom J., Agarwal V., Lilleker J.B., Tan A.L., Nune A., Shinjo S.K. (2023). COVID-19 vaccination in autoimmune diseases (COVAD) study: Vaccine safety and tolerance in rheumatoid arthritis. Rheumatology.

[57] De Stefano L., Balduzzi S., Bogliolo L., D’Onofrio B., di Lernia M., Mauric E., Milanesi A., Brandolino F., Rocca C., Chiricolo S. (2023). Reactogenicity, safety and disease flares following BNT162b2 mRNA COVID-19 vaccine in patients with chronic immune-inflammatory arthritis treated with biological and targeted synthetic disease-modifying anti-rheumatic drugs. Clin. Exp. Rheumatol..

[58] Lee J.J.Y., Bernatsky S., Kwong J.C., Li Q., Kwok T.S.H., Widdifield J. (2024). Safety and Health Care Use Following COVID-19 Vaccination Among Adults with Rheumatoid Arthritis: A Population-Based Self-Controlled Case Series Analysis. J. Rheumatol..

[59] Boekel L., Kummer L.Y., van Dam K., Hooijberg F., van Kempen Z., Vogelzang E.H., Wieske L., Eftimov F., van Vollenhoven R., Kuijpers T.W. (2021). Adverse events after first COVID-19 vaccination in patients with autoimmune diseases. Lancet Rheumatol..

[60] Oleszczyk M., Marciniak Z., Nessler K., Wójtowicz E., Szozda N., Kryj-Radziszewska E., Boroń M., Gajos K., Paziewski M.P., Sajdak P. (2023). COVID-19 vaccine short-term adverse events in the real-life family practice in Krakow, Poland. Eur. J. Gen. Pract..

[61] Machado P.M., Lawson-Tovey S., Strangfeld A., Mateus E.F., Hyrich K.L., Gossec L., Carmona L., Rodrigues A., Raffeiner B., Duarte C. (2022). Safety of vaccination against SARS-CoV-2 in people with rheumatic and musculoskeletal diseases: Results from the EULAR Coronavirus Vaccine (COVAX) physician-reported registry. Ann. Rheum. Dis..

[62] Takatani A., Iwamoto N., Koto S., Aramaki T., Terada K., Ueki Y., Kawakami A., Eguchi K. (2023). Impact of SARS-CoV-2 mRNA vaccine on arthritis condition in rheumatoid arthritis. Front. Immunol..

[63] Ma M., Santosa A., Fong W., Chew L.C., Low A.H., Law A., Poh Y.J., Yeo S.I., Leung Y.Y., Ng V.W.W. (2023). Post-mRNA vaccine flares in autoimmune inflammatory rheumatic diseases: Results from the COronavirus National Vaccine registry for ImmuNe diseases SINGapore (CONVIN-SING). J. Autoimmun..

[64] Rider L.G., Parks C.G., Wilkerson J., Schiffenbauer A.I., Kwok R.K., Farhadi P.N., Nazir S., Ritter R., Sirotich E., Kennedy K. (2022). Baseline Factors Associated with Self-reported Disease Flares Following COVID-19 Vaccination among Adults with Systemic Rheumatic Disease: Results from the COVID-19 Global Rheumatology Alliance Vaccine Survey. Rheumatology.

[65] Striani G., Hoxha A., Lorenzin M., Cozzi G., Scagnellato L., Vangelista T., Frizzera F., De Sandre P., Simioni P., Doria A. (2023). The impact of SARS-CoV-2 infection and vaccination on inflammatory arthritis: A cohort study. Front. Immunol..

