# Supplementary material for: Effectiveness and Safety of the COVID-19 Vaccine in Patients with Rheumatoid Arthritis in a Real-World Setting
Source: Vaccines (Basel). 2024 Jun 18;12(6):672. doi: 10.3390/vaccines12060672 (PMC11209506; doi:10.3390/vaccines12060672)
Supplement: Supplementary file 1 [file vaccines-12-00672-s001.zip › vaccines-3021117-supplementary.pdf]

Supplementary Materials

Table S1. Description of analyzed variables.

Groups of variables	Type of variable	Description
**Sociodemographic variables**	Age	Years
	Sex	Male, female
	Smoking	Never, past or current smoker<br>Pack-years
	Comorbidities	Presence<br>Type: AHT, DM, DL, obesity, ischemic heart disease, thromboembolic disease, cardiovascular disease, stroke, peripheral arterial disease, arrhythmias, heart failure, thromboembolic disease, pulmonary thromboembolism, deep vein thrombosis, renal failure, COPD, asthma
**RA characteristics**	Onset date	Date
	Diagnosis date	Date
	Rheumatoid factor	Presence, titer
	aCCP antibodies	Presence, titer
	Erosive RA	Yes / No
	Extra-articular manifestations	Nodules, interstitial lung disease, atlanto-axoid subluxation, others
	Disease activity and functionality	ESR, CRP, DAS28, HAQ
**RA treatment**	Previous therapy	GC, MTX, HCQ, SSZ, CsA, AZA, MPM, TACRO, TOFA, BARI, ANAK, ABA, RTX, TCZ, SARI, IFX, ETN, ADA, GOLI
	Current therapy	Starting date and dosage of GC, MTX, HCQ, SSZ, CsA, AZA, MPM, TACRO, TOFA, BARI, ANAK, ABA, RTX, TCZ, SARI, IFX, ETN, ADA and/or GOLI
**Previous COVID-19**	Diagnosis	Data, method (PCR / antigen test), place (Primary Care / Hospital Care)
	Symptoms and complications	Cough, fever, expectoration, nasal congestion, pneumonia, dyspnoea, desaturation, chest pain, palpitations, tachycardia, back pain, arthralgia, myalgia, arthritis, malaise, anosmia, ageusia, nausea, vomiting, diarrhoea, headache, dizziness, rash, superinfection, thrombosis, arrhythmia, renal failure
	Severity	Hospital admission, ICU admission
	Sequelae	Free text for description
	RA activity at diagnosis of COVID-19	Remission, low, moderate, severe
	Treatments received	Anti-inflammatory, antiviral, respiratory support, modification of usual RA treatment
**COVID-19 vaccine**	Type of vaccine	AZD1222, Ad.26.COV2.s, mRNA-1237, BNT162b2
	Number of vaccine doses	Number of doses administered
	First dose	Date, AE (fever, arthralgia, myalgia, arthritis, headache, dizziness, nausea, vomiting, rash, anaphylaxis, thrombosis), severe AE

	Second dose	Date, AE (fever, arthralgia, myalgia, arthritis, headache, dizziness, nausea, vomiting, rash, anaphylaxis, thrombosis), severe AE
	Humoral response*	Post-vaccine serology (BAU/mL), serology collection date
**Post-vaccine COVID-19**	Symptoms and signs	Cough, fever, expectoration, nasal congestion, pneumonia, dyspnoea, desaturation, chest pain, palpitations, tachycardia, back pain, arthralgia, myalgia, arthritis, malaise, anosmia, ageusia, nausea, vomiting, diarrhoea, headache, dizziness, rash, superinfection, thrombosis, arrhythmia, renal failure
	Severity	Hospital admission, ICU admission

ABA: abatacept; aCCP: anti-cyclic citrullinated peptide antibodies; ADA: adalimumab; AE: adverse events; AHT: arterial hypertension; ANAK: anakinra; AZA: azathioprine; BARI: baricitinib; BAU: Binding Antibody Units; COPD: chronic obstructive pulmonary disease; COVID-19: Coronavirus Disease 2019; CRP: C-reactive protein; CsA: cyclosporine A; CTZ: certolizumab; DAS28: Disease Activity Score of 28 joints; DM: diabetes mellitus; DL: dyslipidemia; ESR: erythrocyte sedimentation rate; ETN: etanercept; GC: glucocorticoids; GOLI: golimumab; HAQ: Health Assessment Questionnaire; ICU: intensive care unit; IFX: infliximab; LF: leflunomide; mL: milliliter; MPM: mycophenolate mofetil; MTX: methotrexate; HCQ: hydroxychloroquine; PCR: polymerase chain reaction; RA: rheumatoid arthritis; RTX: rituximab; SARI: sarilumab; SSZ: sulfasalazine; TACRO: tacrolimus; TCZ: tocilizumab; TOFA: tofacitinib.

*Anti-SARS-CoV-2 IgG antibody titers (obtained with the SARS CoV-2 IgG II QUANT Alinity technique, Abbott®) were considered negative if <7.1 BAU/mL and positive if ≥7.1 BAU/mL.

Figure S1. Comorbidities of the RA population.

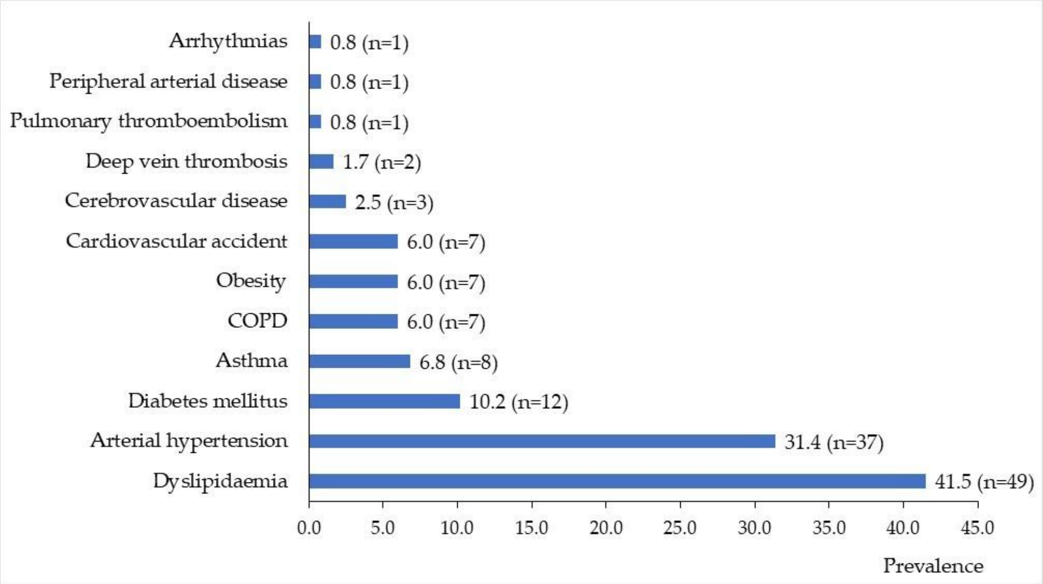

COPD: chronic obstructive pulmonary disease.
Prevalence of comorbidities is expressed as percentage (number of patients).
